# Human Development VIII: A Theory of “Deep” Quantum Chemistry and Cell Consciousness: Quantum Chemistry Controls Genes and Biochemistry to Give Cells and Higher Organisms Consciousness and Complex Behavior

**DOI:** 10.1100/tsw.2006.257

**Published:** 2006-11-14

**Authors:** Søren Ventegodt, Tyge Dahl Hermansen, Trine Flensborg-Madsen, Maj Lyck Nielsen, Joav Merrick

**Affiliations:** ^1^ Quality of Life Research Center, Teglgå rdstræde 4-8, DK-1452 Copenhagen K, Denmark; ^2^ Research Clinic for Holistic Medicine, Copenhagen, Denmark; ^3^ Nordic School of Holistic Medicine, Copenhagen, Denmark; ^4^ Scandinavian Foundation for Holistic Medicine, Sandvika, Norway; ^5^ Zusman Child Development Center, Soroka University Medical Center, Ben Gurion University of the Negev, Beer-Sheva, Israel; ^6^ National Institute of Child Health and Human Development, Jerusalem, Israel; ^7^ Office of the Medical Director Division for Mental Retardation, Ministry of Social Affairs, Jerusalem, Israel

**Keywords:** holistic biology, public health, quantum mechanics, quantum chemistry, Denmark

## Abstract

Deep quantum chemistry is a theory of deeply structured quantum fields carrying the biological information of the cell, making it able to remember, intend, represent the inner and outer world for comparison, understand what it “sees”, and make choices on its structure, form, behavior and division. We suggest that deep quantum chemistry gives the cell consciousness and all the qualities and abilities related to consciousness. We use geometric symbolism, which is a pre-mathematical and philosophical approach to problems that cannot yet be handled mathematically. Using Occams razor we have started with the simplest model that works; we presume this to be a many-dimensional, spiral fractal. We suggest that all the electrons of the large biological molecules orbitals make one huge “cell-orbital”, which is structured according to the spiral fractal nature of quantum fields. Consciousness of single cells, multi cellular structures as e.g. organs, multi-cellular organisms and multi-individual colonies (like ants) and human societies can thus be explained by deep quantum chemistry. When biochemical activity is strictly controlled by the quantum-mechanical super-orbital of the cell, this orbital can deliver energetic quanta as biological information, distributed through many fractal levels of the cell to guide form and behavior of an individual single or a multi-cellular organism. The top level of information is the consciousness of the cell or organism, which controls all the biochemical processes. By this speculative work inspired by Penrose and Hameroff we hope to inspire other researchers to formulate more strict and mathematically correct hypothesis on the complex and coherence nature of matter, life and consciousness.

## INTRODUCTION

”The hard problem” in biology, medicine and psychobiology is the problem of the connection between subjective and objective factors in life: how can a super-advanced, chemical machine like our body give us a conscious state? So, what is chemistry, and what is consciousness, and can these two ever meet? Recently many researchers, for example the team of Penrose and Hameroff being one of the most successful[1,2,3,4], made suggestions based on quantum mechanics, as this branch of physics has many of the qualities that is needed for a theory of consciousness, coherence and non-locality being among the most important. Penrose and Hameroff think that the coherent molecule orbitals of the microtubules and other inter- and extra cellular fibers, including the links from cell to cell of these fibers, is what is needed to give the cell consciousness.

The idea we are going to present in this paper and the central idea in this series of 20 papers about human development is closely related to the Penrose-Hameroff idea. We use what we see as a holistic approach wanting to integrate all the large molecule orbitals of the cells molecules, not only the large fibers. The reason why we think this is a more constructive approach is that we want to make the consciousness of the cell continuous with all events in the cell; we want the consciousness to be connected to global information of the cell, and we want cell-consciousness to give the cell the properties of choice – the choice of moving in a direction, taking a form and size, differentiate, divide etc. We simply regard the cell as a complete little animal, and we want it to perceive and “think” very much as we are able to do it ourselves as human beings. The reason that we insist on such a weird idea is that we see the cell as carrying 99% of the complexity of the large animal; therefore we would love the cell also to provide 99% of the reason why we are conscious and feeling, why we can perceive the color red and other qualia, and why we can intuit our world. The cell is not responsible for our complicated interpretation of reality though our brain, but the colony of cells at large is responsible for the interpretation of the world, not the brain itself, as we have discussed it in our theory of the brain[5,6]. So we need a really smart cell to be able to explain what is going on and we need something very creative and ingenious to explain cell intent, cell memory and all the other things we need to understand, before we will feel satisfied with our theory of holistic biology for holistic medicine.

Consciousness is a term that has been kept as a huge biological enigma in scientific history. In this paper we will try to came with a qualified guess of what consciousness is, using traditionally biological terms to explain the connections that lead to consciousness. We will discuss the term from the genes capability of keeping life going in an interaction with proteins, macromolecules, and quantum chemical equilibriums.

Scientists and religious thinkers have discussed the term consciousness in many forums. Seen in a religious perspective consciousness has been supplied with a kind of spiritual body, that the traditional thinking scientist reject without discussion. By this paper we try to build a bridge between these two different kinds of thinking and to describe consciousness as an integrated capability of the universe that is shared out to each single biological individual at the creation of it. And we do this by describing the consciousness as a function of quantum mechanical activity working in a kind of “symbiosis” by controlling the genetic and biochemical functions that is necessary for a cell or an organism to survive. This “symbiosis”, that gives an organism the capability to “feel” the outer world, we call the consciousness. We describe this as a function that has progressed through evolution, and has been more and more “conscious” with the expansion of the complexity in biological organisms, which in the end has lead to the human consciousness that has been capable of developing the industrialized world.

We have already described the fractal geometry[7] and the super orbitals[7]. In this paper we will look closer at the biochemical activity that leads to the control of master genes, cell volume control, cell proliferation, cell movements, cell communication, cell interactions, etc., to be able to understand the term of cell consciousness, collectively cell consciousness (e.g. organ and tissue consciousness), and consciousness of a complete multi cellular individual.

## From Active Proteins to Energized Global Electron Orbitals

Most of the proteins of the cell use ATP (Adenosine triphosphate) to do their job. When ATP is broken down to ADP and P, a protein is statistically forced into one allosteric shape with the proteins typical power. When it switches back and releases its build-in energy it must be recharged to work again. In this way the proteins are "paid" to dance, and the dance is typically a thing that happens in the molecule orbital, like proton pumping. The cell itself produces the ATP necessary for the cell function. ATP is produced by ATP-synthesis imbedded in the cell surface and through the energetic metabolism in the mitochondrion. This circular quantum chemical self-production of energy thereby, makes the cell able to control its own life cycles by just getting food and liquid supply from outside like a multi cellular organisms that functions like a complete body symbiosis. The connection between energetic supply from outside and quantum mechanical activity can best de illustrated by using the plants photosynthesis as a model. In plants, the sun delivers the energy that is absorbed by photo pigments. Such pigments can have a lot of different colors, e.g. red, green, yellow, blue. Each color represents a specific energetic quantum corresponding to a striked electron in a specific orbit. In this way the plant by itself, can decide the exactly quantum of energy suitable for its needs. The striked electrons then are further transported by co-factors in a process that ends up in creating nutrients for the survival of the plant. Such kind of "cyclic" quantum chemical activity guiding both the energetic building up and breaking down activities of the cell, where the cell gets its nutrients from outside and then manifests all its functions through quantum mechanical activity, is very equivalent to the function of a higher multi cellular organism, and emphasizes the whole idea in our way to think.

If the molecular orbital is connected to a higher system, this system could be energized, through the activity of co-enzymes, as well in these movements allowing the super-orbital of the cell to manage energy that can be used to control the dance of other proteins. There are many ligands modifying the activity of active proteins, and these ligands are more active or more passive as their form shifts; a system that can control the protein activity and the likelihood that a protein is in a specific allosteric configuration is then controlling the activity of the cell.

The dance of the co-factors and the proteins gives much energy to the collective electron orbital; the problem is how information is handled in it. As we discussed in the paper on the cell[8], all its molecules combine to supra-molecular structures; these are often large protein molecules with extremely complex molecule orbitals (highly structured clouds of the electrons of the molecule). Interestingly, these patterns are known to be unstable; most proteins are either catalytic enzymes or structural "building block" proteins and they exist in several forms or states, often at least one active and one passive state. So basically life is regulated though the regulation of its proteins. And the proteins are all the time changing their state because of the heat-vibrations, so a small change in the likelihood for one state of the protein at one location in the cell is really what we are looking for. As we are here deeply into quantum chemistry and nobody yet have been able to calculate what is happening at these levels, all we can do is guess.

## "Deep" Quantum Chemistry: The Cell has a Deeply Structured Integrative Quantum Field

It is well known from quantum mechanics that all electrons that are contained in one system are inseparable from each other. In a metal this gives the quality of an "electron gas" responsible for the metallic quality. In water the thermal movements of the water molecules disrupts so much that the collective qualities of the electrons is almost lost. In a cell the cytoplasm is a gel made of about 40% proteins, and the structure of this gel is very much like a liquid crystal; there are collective properties of the electrons but they are difficult to identify and map. The reason why collective properties of electrons in large molecules like proteins and protein ensembles have been difficult to explore is the difficulty describing the collective high-level quantum chemical phenomena mathematically. Actually calculating the collective behavior of the electrons in molecules much larger than water has been almost impossible.

Our approach was mathematical in the beginning, then more symbolic geometrical as it is today. Geometric symbolism is a pre-mathematical and philosophical approach to problems that cannot be handled mathematically (at this moment). What we have done is to guess on the “natural” and neutral structure of electrons, if nothing disturbed them. In metal we have the crystal grid and the nuclei to cause severe disturbances of the energetically pattern a gas of completely free electrons would take. In water we have the water molecules disturbing the pattern. In plasma, we actually see very much the same patterns as in a free gas of electrons, but strong magnetic fields necessary to hold the plasma always gives a strong artificial structure of the electron gas, and the density from liquidized nuclei also gives some deviation from a gas of free electrons.

So the way we precede is simply to guess that the pattern of the free electrons are able to give the cell its non-local qualities. The cell needs as we have seen to have a system that provides information in a dispersed non-local way. And the only place in physics we can hope to find such a phenomenon is in quantum mechanics. Quantum mechanics also got irrational numbers (i) in its formalism[9], the same number that is a part of the mathematical structure of some of the really deep fractals like the Mandelbrot fractal. Therefore we are drawn to quantum mechanics; but the normally used formalisms of quantum mechanics do not have a structure usable for explaining living systems. So we need to find a mathematical or a symbolic geometrical language, which is both compatible with the structure of quantum mechanics and with the structure of life. Such a theory need to be a “hidden variable” theory[10] as there must be some hidden layers and structures there.

Using Occam’s razor we must start with the simplest model that works; we suspect this to be the spiral fractal[7]. So in this paper we investigate some of the features of cells that can be explained if we presume that: all the electrons of the molecular orbitals of all the protein molecules and co-enzymes of the cell, including all the many small molecules embedded in these large biomolecules, and co-factors transporting electrons in the mitochondrion’s and mediating the photosynthesis in plants are making up one huge structure (“a cell-orbital”), which in its structure is as close to the neutral spiral fractal as possible, as this is the structure that the electron field would take if nothing were disturbing it.

## Dissipative Structures

A simple suggestion is by dissipative structures, which is self-organizing patterns arising in systems with a high e-flux. The problem with such patterns is that if they are to be used by biology it must be possible to build on them to make the structure of the cell form and develop. Unfortunately all dissipative structures are bound by the lack of precision of thermal processes like Brown’s movements. So they are not precise enough to build on as the natural tendency is to loose structure and information according to the laws of thermodynamics. The patterns might be there, providing the cell with a lot of creativity and a whole “menu” of patterns for use for its internal structures and its external behaviors; but the cell still needs to be highly structured levels of information in the system to direct the use, enhancement and repression of such patterns.

## Iterative Generators and Fractal Patterns

As the cell is obviously structured by some kind of fractal principle[8] it would be nice to have a phenomenon giving such fractal structures. We know that more complicated fractals are made by iteration of a sometimes extremely simple formalism, like the Mandelbrot fractal. But what in the cell can give that?

The spiral fractal needs some kind of walking impulse to function as a calculator, and if we presume that it sometimes collects this kind of impulse, or if it carries and does this in a self-reinforcing way then we can have the spiral fractal generating all kinds of such patterns.

The interesting thing of such patterns is that it really can be active at any level of detail, and in this way it can control all “local spots” in the cell, including the activity of all its protein molecules, and maybe even modify the quantum state of other, smaller molecules, like fat and sugar – and even the water of the cell.

## What is Cell Consciousness in Biochemical Terms?

Cell consciousness is a very diffuse term that is hard to understand because we not yet have a usable explanation for it. Here we will try to explain the term from a biochemical point of view. To satisfy this term we need to fulfill a number of factors. The cell itself has to function as a complete “symbiosis” including all functions happening in the cell itself controlled from the quantum chemical super orbital. By changing conformation lots of proteins, guided by the energetic quanta supplied from the super orbital, implement countless processes in the cell, leading to important cell functions like e.g. volume control, control of cell cycle, control of cell proliferation, control of cell movement, etc.., but maybe most important, control of master gene and master regulatory protein activity, necessary to support all these processes. It is the gathered quantum mechanical energies standing behind and very strict controlling all this activity in a cell, gathered in a “super” functional energetic “symbiosis”, that we call cell consciousness.

## What is Collective Consciousness and Consciousness of a Multi Cellular Individual in Biochemical Terms?

In this paper we will expand the term consciousness also to count for collectively consciousness. Such term can be explained through the activity of fractal geometry. Through the fractal geometry[7] it is possible to gain collectively control over an amount of cells that are organized in a “social society” through the development of an organ. And again the organs are organized in tissues through social activity that again organizes within a developing organism through embryogenesis and ontogenesis[8,11] forming a full developed adult organism existing of all the body cells in one complete symbiosis. If we accept that energetic quantums[12] are supported by the quantum mechanical super orbital of the individual organisms (an organism, seen as e.g. a cell, an organ, a tissue or a complete organism including all cells) to the structure of fractals, the term of collectively consciousness can be explained by gathering all super orbital energy in the fractal structures, where the fractal structure is responsible for the distribution of the energy. In this way the energetic information (positional information) to organize the body structures as e.g. organs, tissues, whole organisms (in the principles all structures up to complete universes), can be controlled through the fractal geometry. Simply, the energetic super orbital from a single cell represents one level of the fractal. The super orbital energy from several cells delivers the information to the organization of an organ by gathering all super orbital energies from cells involved in the organ development, at the next level of the fractal and so on. Each time a new cell enter into the organ through ontogenesis, the new cells super orbital energy is supplied to the fractal level responsible for delivering the information for the development of the specific organ. This strengthens the united energy of the fractal level, gradually as the complexity of the organism increases. This is going on until a complete adult individual has been developed, gathering all super orbital energies from the single body cells to one very strong super orbital, in humans giving the potential to human consciousness.

Consciousness involves a lot more than this. All biochemical functions and activities in all the cells of the body are involved as described above for the single cell. Gaining collectively consciousness in particular, the cell-to-cell protein interactions, hormonal and other ligands to protein interactions (cell communication) are important, as these factors are absolutely necessary for the cells of a body to communicate.

## How is Inter Cellular Control Gained by the Single Cell?

When a protein guided by the super orbital through strict energetic control change conformation, this causes multiple activity in the cell. Some of the most important processes are listed below.

### *Control of Cell Volume*

A very important function for the single cell to gain control over itself and thereby to exist at all is its capability to control its own volume and being able to regulate its balance of liquids. To gain this control, the cell needs energy delivered by the super orbital through co-enzyme transport and ATP. This energy is used for configuration changes of proteins as Protein Kinase C (PKC) that is placed in the cell membrane and causes transport of ions through the cell membrane[13]. Such function controls the gathered energy balance of the cell through ion balance, and is, following the holistic paradigm, a part of the united amount of quantum mechanical energy that makes up the super orbital. Such transport happens also through channels shaped by proteins changing conformation through use of ATP[14]. This means that the final energetic balance is a part of and strictly controlled by the super orbital.

### *Control of Master Genes and Master Regulatory Proteins*

How do the fractal positional information and the super orbital gain control with the DNA and the important proteins of the cell? The processes gaining control with the proteins DNA helicase and DNA topoisomerase, controls the unwinding and the super-coiling of DNA respectively[14]. These processes are essential for cell activity, because it is involved both in control of cell proliferation (described under) during DNA replication, and the control of DNA transcription. Transcription of DNA leads to production of proteins of which some again are involved in control of the DNA activity (master genes and master regulatory proteins). Such processes make up the “heart” of the living cell and are under strict control from the super orbital that supports exactly the right amount of energy to make a precise regulation of a cells DNA expression. This means that involved in this process a strict control of ligands (master regulatory proteins) also have to be governed from the super orbital. This is a kind of “feed back” process, where genes produce ligands that control the DNA expression[14]. Such processes are essential to gain consciousness, because DNA activity is necessary for production of new cells, and thereby the creation of several new super orbitals.

### *Control of the Cell Cycle*

The cell cycle is essential to gain control over the cell proliferation[14,15], and thereby the amount of cells participating in the formation of a multi cellular body as e.g. an organ, a tissue or a complete body. This cycle is under strict genetic control and under strict control from master regulatory proteins as e.g. the E-box controlling proteins[16]. Following the holistic paradigm these processes are again firmly controlled from the super orbital through control of protein configurations and support of ATP. This means that these processes are under stringent control from the consciousness.

### *Control of Cell Proliferation*

Cell proliferation is a process that is firmly coupled to the cell cycle[14,15]. These two processes together determine how many cells a multi cellular organism shall contain. Cell proliferation includes processes as control over the microtubule involved in separation of the chromosomes through cell proliferation, control over the split up of the cell membrane, the cytoplasm and the organelles, and control over the gathered cytoskeleton[15]. These processes have to be governed in a very precise and strict process, where everything following the holistic paradigm is guided through the super orbital that support the precisely right amounts of energies necessary for these processes to take place. This means that this complicated cellular symbiosis is formed by lots of incredible precisely guided processes all necessary to gain cell consciousness through the production of new cells.

### *Control of Cytoskeleton Activity Including Cell Movements*

Cell movements[8] are a consequence of the ability to control the cytoskeleton. Control of the cytoskeleton is essential for the cell to gain contact with the extra cellular world, as it is an important factor of the cell proliferation as mentioned above. The ability to move has evolved through evolution from the amoeba that crawls slowly and feels in which direction it will move[8], to cells as single cell algae that has developed flagella to control its movements[15]. Control of such senses in the single organism and development of these capabilities through evolution, is following the holistic paradigm under strict control from the super orbital and is a part of the gathered qualities that makes the consciousness through quantum chemical activity.

## How is Intra Cellular Control Gained between Cells and Multi Cellular Bodies?

### *Control of Cell Communication through Hormone and Ligand Activity*

To gain collective control of a multi cellular body as e.g. an organ, a tissue or a complete multi cellular organism, it is essential to be able to communicate between all cells participating in the formation of the body. To gain such control, we have already mentioned that the fractal organization of different fractal levels, where the energetic distribution of super orbitals is essential for our model to explain collectively cell communication. But this is not enough in itself. Biochemical activity under strict control from the super orbitals of the single cells of the body, or from the super orbitals of the single bodies of a complete multi cellular organism as e.g. the human body, is following the holistic paradigm, also essential to create a collective consciousness. Such intra cellular communication is managed by genetic control through production of hormones and other ligands[15]. Such proteins are secreted onto the extra cellular liquids and distributed between cells. Hormones and other ligands bind to protein receptors of the outer cell surface, and change the conformation of these receptors[15]. Such activity entrepreneurs huge amounts of activity, that both are involved in development (growth hormones), gene regulation, cell volume control, etc.[15]. Such activities are essential to gain collectively consciousness of a multi cellular body, and for multi cellular bodies to gain collectively consciousness of a complete organism. In e.g. humans, the complexity of the gathered super orbital is so powerful and strong, that it has caused the development of “through” consciousness, leading to the creation of the industrialized world.

### *Control of Cell Communication through Cell-to-Cell Adhesion*

Another way for the cells to communicate directly with each other is through cell-to-cell adhesion[15]. This involves protein interactions between proteins placed directly on the surface of the cell. In this way the cells are gathered in everything from clusters of two cells, to clusters of a huge amount of cells. This kind of communication also involves the change of confirmation of the proteins involved in the cell-to-cell adhesions, and thereby the delivery of ATP energy governed from the super orbital of the single cell and from the gathered super orbital of the multi cellular body. Such processes are also following the holistic paradigm an important part of the collective body consciousness, and participate in the "gathered" consciousness of the complete multi cellular organism.

## The Cell's Representation of the World

It is well known that the cell is attentive towards both its interior and its exterior world[15]. But how is it representing the world, and how is it interpreting what it "sees"? The spiral fractal gives a simple solution to the problem of representation; because the nature of the spiral is that it is like a spring that becomes vibrating in a specific pattern mirroring the phenomenon that impacted it. In this way the spring's cellular level will mirror both its inner and outer world.

## The Cell's Coherence

The mutual representation of cells makes cells cohere; they share a common reality and the whole cellular system is sharing almost the same information. Of cause will small differences like being close to the organisms borders give a little different clues to the cells that being in the middle.

## The Cell's Remembering

Patterns wandering up and down the levels of the cell are a simple way to explain cell memory, both remembering and storing. The remembering is guided by an associative principle, as is the storage.

## The Cell's Intent

Inherited patterns deeply embedded in the deep structure of the cells quantum field will serve as intent, giving a constant pressure on the cell to identify useful structures and situations and materializing the patterns corresponding to the inherited patterns. This is the simplest way to describe intent, it seems.

## How the Cell Thinks

Now, can the cell think? Well, it seems that is can perform extremely complex calculations in the collective quantum field of all its electrons, judged from the symbolic geometry of the spiral fractal[7]. If one imagines that the cell has an energetic impulse rotating in its border circle, then this walking impulse can be used for a kind of primitive thinking.

## The Cell's Perception

Now, will the cell be conscious? It depends on how you define consciousness. If you define it as ability to represent and use the representation for making meaningful choices, you must ask if a global representation can be meaningful and if it can lead to choices. It seems from the symbolic geometry that the cell might even be very conscious, and that it can take decisions.

## The Cell's Choices (to Take Structure, Form and Size, Move or Stay, Differentiate, and Divide)

A choice must be taken whenever there is several options to chose from. The cell will identify different possibilities that match its intents, and the preferred pattern must be picked. If all the "urges" of the cell is available through internal representation and all the possibilities of the external world are represented by external representation it is very simple for the cell to make its choice. It can move or stay, it can divide or not, it can differentiate or not.

## The Collective Consciousness of the Cells

The deeply structured quantum field of a cell will resonate well with the quantum field of the other cells, giving the colony of cells collective consciousness. Higher order structures can then materialize in the colony making the cells building blocks of the organism. In the same way as the cell controlled its internal parts, the huge colony of cells making the organism is able to control all its organs and cells. The collective consciousness of the cell is the informational backbone of the organism, and the organism's consciousness raises at the top level of this highly ordered hierarchy.

## Cell's Morphogenesis

The spiral fractal and the deep quantum chemistry seem to provide the cell with many of its mysterious vital functions. It can remember and perceive, it can intent, make choices and move. This is what it takes for the cell to assume whatever forms necessary for it self and, if social, also to serve the colony it is a part of; by using these abilities in a community of cells they can be responsible for morphogenesis of the whole organism. Many cells have morphogenesis; if cells need to change their diet, they can change their internal body structure, building a completely new body with the same molecules as before[8]. Interestingly this also seems to demonstrate that cellular form is not a product of genes but of information bound to the "deep field" of the cell.

## Metamorphosis

Metamorphosis seems to be the most difficult problem to explain: how can a colony of cells suddenly decide that they will shift the form of the total being? But if the morphogenesis is a part of the inherited memory, then metamorphosis will happen as soon as the possibilities of world and the collective consciousness of the organism's cells invites this to happen. All the cells will see that now is the time to materialize this more evolved pattern, and they will chose to go that way.

## How is Human Consciousness Gained?

We think that "Metamorphous Top down" evolution[17] has evolved through consciousness, but human consciousness has developed through "Metamorphous Top down" evolution. E-box master genes that govern the circadian clock feed back loop are essential for the process of metamorphosis in all organisms from cyanobacteria to humans[16]. This indicates that processes governed by the super orbital direct the process that stands behind metamorphosis in all organisms from bacteria to humans. Following the holistic paradigm such genes and other master genes involved in metamorphosis are governed by the super orbital, and thereby that consciousness stands behind metamorphosis. But through evolution, individuals so complex as humans have gained such a huge and powerful gathered super orbital, that it has given the evolution the possibility to create the human consciousness through “Metamorphous Top down” evolution.

## How can Repressed and Disturbed Consciousness of Man Lead to Disease and Death?

All the activity described in this paper so far, gives the feeling of a very complicated and fine regulated mechanism, leading to the term of consciousness. But, how can all these fine tuned processes always lead to fitness and survival? The fact is that they do not. Not much mismatch is necessary for malfunctions to disturb the wholeness. How then can such malfunctions arise? We think that malfunctions leading to disease and death are created by errors in the distribution of orbital energy through the fractal levels, leading to wrong positional information for the process of ontogenesis through development and to the maintenance of an adult body. This is guided by a very fine interaction between the intension of the consciousness, disturbing the patterns that shape our pictures of the reality. Thereby, we ourselves transfer a wrong or “sick” picture of the reality to the fractals that again govern a “mal” distribution of the informational energetic particles of the different fractal levels, leading to disease and death. People that suffer from such malfunctions are maybe mentally disabled and need to be guided in the right direction of a holistic medical practitioner. Following the holistic paradigm such malfunctions can be recovered by guiding a patient to look at the world in a positive way and see all the positive things in itself, face the evil inside itself and repel it. It can regulate the distribution of energetic particles of a fractal level in a way so the information for the correct household of the human body is corrected, leading to fitness and survival. In other words, we believe that a human is its own best medicine; just it is guided in the right direction this will lead to self-recovery through adult human metamorphosis[18].

## DISCUSSION

The presented theory of deep quantum chemistry, together with the simple structure of the spiral fractal, seems to give us a perceptive, conscious and thinking cell, creating its own world though intent and the use of historically collected information. There are many problems not solved with this model. If the structure is really infinite, why will information ever return to the here and now “surface” of reality? Why will it not just travel inwards and outwards in this structure forever and ever? How can the transport of information be so fast – almost instantaneous? We need something traveling much faster that the speed of light to explain consciousness it seems, and if everything is so speedy in this structure how can we have events in real time? And if this “highly structured collective quantum field” is really so powerful, why is it that we cannot read each other’s minds much better? Actually this theory should make us so powerful that we all should be like prophets, commanding the fig tree to die, and thus just killing it or commanding the sick to stand up and be cured. As usual with such theories it is very difficult to make a powerful suggestion and not make it too powerful. The empowerment from this theory means that we should be able to remember everything that ever happened, since beginning of life on the planet; the intense coherence means that we also should know all that happens at the planet just now, and these two combined means that we should be able to remember all that ever happened on the planet. This is not our daily understanding of our human potentials; this is the reality of the wildest shaman and the wisest of Indian sages. And maybe we are all meant to know it all, to be divine, to be enlightened… Maybe this theory on deep quantum fields of information is true, and we are still evolving as humans towards our final destination. We understand fully if the reader finds our suggestion fantastic and too simplistic at the same time.

In the clinic we often have the strange experience of knowing much more about a patient that should be possible, if information was carried by our senses only. It is as if our bodies are reading the other person as this person contacts his or her old traumatic life event, and often the feeling of what happened and even the pictures and sentences of the event is clear to the therapist long before it re-surfaces into the consciousness of a patient. This sharing of information in coherent fields of humans are well known from Tibetan Buddhism, where many monks meditate on the same object, which they all visualize but from the angle of space where they are sitting. So nobody sees the same picture; all sees the same object. The Dalai Lama told us in 1996 (personally communication) that one of his friends could make a knot on a hard needle when meditating, softening the metal with his consciousness. And the Engineering Anomalies Research has for many years conducted research confirming the ability of human consciousness to interact powerfully with even dead objects like computers[19]. To explain such phenomena the presented hypothesis seems also appropriate.

We are at the edge of science, the place where science meets fantasy and imagination. We have dreamt a dream of the inner mechanics of life, and it explains so much that we must share it, for other people to dream it forward to. And one day we might even be able to test the existence of deeply structured biological quantum fields in the laboratory.

It is the collective control of all the mentioned activities and all these functions of the single cell and many interacting cells in symbiosis that together involves the term consciousness regardless if it is the consciousness of a single cell, an organ, a tissue or a complete multi cellular organism. The consciousness is a symbiotic control of all energies, gathered in a wholeness, creating a superior super orbital of quantum chemical activity, regulating the whole pattern of behavioral activity necessary for survival, including all genetic and biochemical activity of a cell or a multi cellular body, whatever it applies to a single cell or a multi cellular body of higher levels during the ontogenesis, or it applies to a mature or adult organism. We think that consciousness is a function of energies that exists over all in the universe created by the energetic equilibrium that is the result of everything. The consciousness of a biological organism is only a body in the universe that has “borrowed” some of the gathered energy of the universe to gain its own consciousness gathered in its own “little” closed orbit. Seen in this perspective, all elements and components of the universe could be described as having its own consciousness. We believe that consciousness of biological organisms exists in self the most primitive organisms on earth as e.g. the bacteria. And it has existed in all organisms from the first living cell was created in the primordial soup. The consciousness of all living organisms is just borrowed from the gathered consciousness of the universe (God). We believe that consciousness in biological organisms has evolved through the evolutionary history as it gained more and more “nearness” with the surrounding world as the complexity of multi cellular organisms evolved. With the human being, this “nearness” became so revealed through the capability of independent mentality, that it was capable of developing the industrialized world. We think this development has evolved through a process we call the “Metamorphous Top down” evolution. The human being has gained so strong control over its consciousness that it is capable of focusing itself through ordering a mal-distribution of the energetic information of a fractal structure that maintains the organism in a state of sickness and psychical disturbance, in a way so it again can be fit and good for survival and capable of doing this by itself without any needs of medical products. It does this through control of master gene activity during adult human metamorphosis, that is the human’s own medicine. However, sometimes it is necessary to be helped to think the right way by a holistic medical practitioner. The human has gained all these capabilities through the development of human consciousness. This means that human consciousness has been developed through “Metamorphous Top down” evolution, while “Metamorphous Top down” evolution has been implemented by consciousness through the evolutionary history.

## CONCLUSION

We suggest that the cell has consciousness and all the qualities and abilities related to consciousness. Geometric symbolism is a pre-mathematical and philosophical approach to problems that cannot be handled mathematically (yet). What we have done is to guess on the “natural” and neutral structure of electrons if nothing disturbed them. So the way we precede is simply to guess that the pattern of the free electrons are able to give the cell its non-local qualities. Using Occam's razor we must start with the simplest model that works; we suspect this to be the spiral fractal[7]. So in this paper we investigate some of the features of cells that can be explained, if we presume that all the electrons of the molecular orbitals of all the co-factors and protein molecules of the cell, including all the many small molecules embedded in these large bio-molecules are making up one huge structure ("a cell-orbital"). Its structure is as close to the neutral spiral fractal as possible, as this is the structure that the electron field would take if nothing were disturbing it.

Most of the proteins of the cell use ATP to do their job. When ATP is broken down to ADP and P, a protein is statistically forced into one allosteric shape with the proteins typical power. When it switches back and releases its build-in energy is must be recharged to work again. In this way the proteins are "paid" to dance, and the dance is typically a thing that happens in the molecule orbital, like proton pumping. Even the "payment" is administrated by the cell itself, delivered through co-factors that build up the ATP molecules through quantum chemical activity.

If the molecular orbital is connected to a higher system, this system could be energized as well in these movements allowing the super-orbital of the cell to manage energy that can be used to control the dance of other proteins. There are many ligands modifying the activity of active proteins, and these ligands are more active or more passive as their form shift; a system that can control the protein activity and the likelihood that a protein is in a specific allosteric configuration is then controlling the activity of the cell. The dance of the proteins gives much energy to the collective electron orbital; the problem is now on a more detailed level how information is handled in it.

Consciousness of biological organisms is the capability of gaining full control over all aspects of life through the super orbital, by controlling all energetic activity of a cell or a multi cellular body, leading to full control of all biochemical activity in a single cell or a multi cellular body, in a way so all activity of the body interacts in a functional symbiosis. We think that consciousness is a main activity of the universe naturally assigned to all biological organisms when these are created. We believe that consciousness has evolved through the evolution from bacteria to humans, and in humans it has been so focused against the outside world that they have evolved the capability of thinking. We think this capability has evolved through "Metamorphous Top down" evolution that has given the human being the capability, through adult human metamorphosis, to gain self recovery from diseases and psychical disorders.

## ACKNOWLEDGMENTS

These studies were supported by grants from IMK Almene Fond. Our research in quality of life has been approved by the Copenhagen Scientific Ethical Committee under number (KF)V.100.2123/91.

## References

1. Penrose, R. (1990) *The Emperor's New Mind*. Oxford University Press.
2. Stuart, R., Hameroff, S.R., Kaszniak, A.W., and Scott, A.C. (1996) *Toward a Science of Consciousness: The First Tucson Discussions and Debates (Complex Adaptive Systems).* The MIT Press.
3. Stuart, R., Hameroff, S.R., Kaszniak, A.W., and Scott, A.C. (1998) *Toward a Science of Consciousness II: The Second Tucson Discussions and Debates (Complex Adaptive Systems)*. The MIT Press.
4. Stuart, R., Hameroff, S.R., Kaszniak, A.W., and Scott, A.C. (1996) *Toward a Science of Consciousness III: The Third Tucson Discussions and Debates (Complex Adaptive Systems).* The MIT Press.
5. Ventegodt, S., Andersen, N.J., and Merrick, J. (2003) Quality of life philosophy IV. The brain and consciousness. *TheScientificWorldJOURNAL* **3**, 1199–1209.
6. Ventegodt, S., Hermansen, T.D., Flensborg-Madsen, T., Clausen, B., Nielsen, M.L., and Merrick, J. (2006) Human development XII: a theory for the structure and function of the (human) brain. Submitted to *TheScientificWorldJOURNAL.*
7. Ventegodt, S., Hermansen, T.D., Flensborg-Madsen, T., Rald, E., Nielsen, M.L., Clausen, B., and Merrick, J. (2006)

Human development VII: a spiral fractal model of fine structure of physical energy could explain central aspects of biological information, biological organization, and biological creativity. *TheScientificWorldJOURNAL* **6**, 1434-1440.

8. Hermansen, T.D., Ventegodt, S., Clausen, B., Nielsen, M.L., and Merrick, J. (2006) Human development IV: the living cell has information-directed self-organization. *TheScientificWorldJOURNAL* **6,** 1132–1138.
9. Feynman, R.P., Leighton, R.B., and Sands, M. (1971) *The Feynman Lectures on Physics: Commemorative Issue. Vol 1. Mainly Mechanics, Radiation, and Heat*. Addison Wesley.
10. Bachtold, A., Schonenberger, C., Strunk, C., Salvetat, J.P., and Forro, L. (1999) *Aharonov-Bohm oscillations in multiwalled carbon nanotubes*. NANOTUBE-99 Workshop.
11. Ventegodt, S., Hermansen, T.D., Flensborg-Madsen, T., Clausen, B., Nielsen, M.L., and Merrick, J. (2006) Human development VI: supracellular morphogenesis. The origin of biological and cellular order. *TheScientificWorldJOURNAL* **6,** 1424-1433.
12. Ventegodt, S., Hermansen, T.D., Rald, E., Flensborg-Madsen, T., Nielsen, M.L., Clausen, B., and Merrick, J. (2006) Human development III: bridging brain-mind and body-mind. Introduction to “deep” (fractal, poly-ray) cosmology. *TheScientificWorldJOURNAL* **6,** 767–776.
13. Hoffmann, E.K. and Dunham, P.B. (1995) Membrane mechanisms and intracellular signalling in cell volume regulation. *Int. Rev. Cytol.* **161**, 173–262.
14. Zubay, G.L. (1998) *Biochemistry*. Wm. Brown Publishers, Dubuque, IA.
15. Alberts, B., Johnson, A., Levis, J., Raff, M., Roberts, K., and Walker, P. (2002) *Molecular Biology of the Cell*. Garland Science, Taylor & Francis Group.
16. Hardin, P.E. (2004) Transcription regulation within the circadian clock: the E-box and beyond. *J. Biol. Rhythms* **19(5)**, 348–360.
17. Hermansen, T.D., Ventegodt, S., Clausen, B., Nielsen, M.L., and Merrick, J. (2006) Human development X: explanation of the macro-evolution: top down evolution materialises consciousness. The origin of metamorphosis. Submitted to *TheScientificWorldJOURNAL*.
18. Ventegodt, S., Hermansen, T.D., Clausen, B., Nielsen, M.L., and Merrick, J. (2006) Human development XX: adult human metamorphosis. Submitted to *TheScientificWorldJOURNAL*.
19. Jahn, R.G. (1982) The persistent paradox of psychic phenomena: an engineering perspective. *Proc. IEEE* **70(2)**, 36–170.
